# Myeloid DJ-1 deficiency protects acetaminophen-induced acute liver injury through decreasing inflammatory response

**DOI:** 10.18632/aging.203340

**Published:** 2021-07-21

**Authors:** Bingrui Wang, Jichang Li, Junzhe Jiao, Min Xu, Yichun Luo, Fang Wang, Qiang Xia, Yueqiu Gao, Yu Feng, Xiaoni Kong, Xuehua Sun

**Affiliations:** 1 Department of Liver Surgery, Renji Hospital, School of Medicine, Shanghai Jiao Tong University, Shanghai, China; 2 Central Laboratory, Department of Liver Diseases, ShuGuang Hospital Affiliated to Shanghai University of Chinese Traditional Medicine, Shanghai, China; 3 Department of General Surgery, Shuguang Hospital, Shanghai University of Traditional Chinese Medicine, Shanghai, China

**Keywords:** acetaminophen, acute liver failure, inflammation, reactive oxygen species, DJ-1

## Abstract

Background: DJ-1 (also known as PARK7), a noted protein implicated in modulating ROS production and immune response, has been observed to play critical roles in the pathogenesis of many forms of liver disease through multiple mechanisms. However, its role and specific mechanism in acetaminophen (APAP) -induced liver injury have not been explored.

Results: In this present study, by employing an acute liver injury induced by APAP overdose mouse model, we demonstrated that DJ-1 knockout (DJ-1^−/−^) mice showed reduced liver injury and lower mortality. In accordance with these changes, there were also alleviating inflammatory responses in both the serum and the liver of the DJ-1^−/−^ mice compared to those of the wild-type (WT) mice. Functional experiments showed that APAP metabolism did not affected by DJ-1 deficiency. In addition, to investigate DJ-1 modulates which kind of cell types during APAP-overdose-induced acute liver injury, hepatocyte-specific DJ-1-knockout (Alb-DJ-1^−/−^) and myeloid-specific DJ-1-knockout (Lysm-DJ-1^−/−^) mice were generated. Interestingly, hepatic deletion of DJ-1 did not protect APAP-overdose induced hepatotoxicity and inflammation, whereas Lysm-DJ-1^−/−^ mice showed similar protective effects as DJ-1^−/−^ mice which suggest that the protective effects of deletion of DJ-1 was through modulating myeloid cell function. Consistently, there were alleviated pro-inflammatory cells infiltration and reduced reactive oxygen species (ROS) production in the liver of Lysm-DJ-1^−/−^ mice relative to control mice.

Conclusion: our findings clearly defined that deletion of DJ-1 protects APAP-induced acute liver injury through decreasing inflammatory response, and suggest DJ-1 as a potential therapeutic and/or prophylactic target of APAP-induced acute liver injury.

## INTRODUCTION

Acetaminophen (APAP) is a drug used worldwide for treating antipyresis and analgesia and was considered to be safe and effective, but acute liver necrosis following APAP overdose has been observed as early as 1966 [1]. Over the past few decades, APAP overdose induced liver injury has become the uppermost cause of acute liver failure (ALF) in developed nations, which ultimately result in liver transplantation [2–6]. Given concerns caused by the high incidence and severity of APAP-induced hepatotoxicity, there have been extensive studies aiming to understand the mechanisms of acetaminophen-induced liver injury. Although APAP-induced oxidative stress and mitochondrial dysfunction have been considered to be the first and foremost event in APAP-induced liver injury [7], there are multiple phases and pathways involved in APAP hepatotoxicity, including oxidative stress, APAP metabolism, endoplasmic reticulum (ER) stress, sterile inflammation and liver repair [8–11]. Among these, hepatotoxic cascade was considered to be initiated by mitochondrial protein adducts generated by APAP metabolism, which caused increased mitochondria oxidant stress and consequently led to mitochondrial dysfunction, necrosis of hepatocytes, and the leakage of damage-associated molecular patterns (DAMPs), including nuclear DNA, HMGB1 and mitochondrial DNA. DAMPs subsequently activate proinflammatory cells to infiltrate and release cytokines, which further result in aggravating liver injury. Therefore, APAP metabolism, mitochondrial dysfunction, and proinflammatory responses are three consecutive adverse incidents of APAP-induced hepatotoxicity.

DAMPs from injured hepatocytes initiate the recruitment and activation of innate immune cells, such as macrophages and neutrophils, which have been shown contributed to the progression of liver injury [12–15]. Activation of immune cells, including the inflammasome complex formation, resulting in the secretion of numerous proinflammatory cytokines, such as interleukin-1 and tumor necrosis factor-α (TNF-α) [16, 17]. The sterile inflammation is likely to intensify the initial assault and enhance overall tissue damage especially in the earlier phase. Among the infiltrated inflammatory cells, the M2 macrophages are critical in liver regeneration. The M2 macrophages generate IL-10 and other cytokines, which contribute to alleviate inflammation and facilitate tissue repair [18]. Furthermore, apoptosis of neutrophils can be induced by M2 macrophages, which may lead to the resolution of the inflammatory response following tissue injury [19]. M1 macrophages are pro-inflammatory macrophages [20]. Generally, M1 macrophages support Th1 polarization of CD4+ T helper cells by providing IL-12 [21]. Given the duplex effects of inflammation in APAP-induced hepatotoxicity, mild inflammatory responses may result in tissue recovery and hepatocytes regeneration, yet excessive inflammatory responses could exacerbate liver injury.

DJ-1, also known as Parkinson disease protein 7 (Park7), was primitively found to be an oncogene cooperatively transformed with Ras [22]. Nowadays it has been considered as a multifunctional protein involved in various diseases, not only in early-onset Parkinson’s disease, but also in many inflammatory diseases and liver dysfunction conditions [23]. Previous studies of our lab have demonstrated that DJ-1 is essential in basal ROS generation through binding to P47^phox^ and critical inactivation of innate immune cells [24]. By impairing hepatic progenitor cell (HPC) - related fibrosis and inflammatory niches formation, DJ-1 deficiency could adversely regulate proliferation of HPCs [25]. In addition, DJ-1 deficiency alleviates liver fibrosis by inhibiting ROS production and inflammation in the liver [23]. Moreover, DJ-1 improves hepatocellular carcinoma cell (HCC) proliferation, thus improves tumorigenesis through regulation of hepatic inflammation [26], and DJ-1 deficiency could enhance fatty acid oxidation to protect from hepatic steatosis [27]. Therefore, DJ-1 is involved in liver injury, inflammation, regeneration, and oncogenesis. Although DJ-1 has been proved to inhibit ROS production and inflammation, the function and mechanism of DJ-1 in APAP metabolism and inflammation during APAP-induced acute liver injury have not been explored yet.

In this present study, by employing an acute liver injury mouse model induced by APAP overdose, we demonstrated that DJ-1 knockout (DJ-1^−/−^) mice showed reduced liver injury and lower mortality. In accordance with these changes, there were also alleviating inflammatory responses in both the serum and the liver of the DJ-1^−/−^ mice compared to those of the wild-type (WT) mice. Further, we found that DJ-1 does not contribute to the APAP metabolism, and hepatic inhibition of DJ-1 expression did not protect from APAP-overdose hepatotoxicity and inflammation, whereas Lysm-DJ-1^−/−^ mice showed similar protective effects as DJ-1^−/−^ mice, which suggest that the protective effects of deletion of DJ-1 was through modulating myeloid cell function. Consistently, there were alleviated liver pro-inflammatory cells infiltration, cytokines expression and reduced reactive oxygen species (ROS) production in the liver of Lysm-DJ-1^−/−^ mice compared to the control mice. These findings suggested that deletion of DJ-1 protects from APAP-induced acute liver damage by decreasing inflammatory responses.

## RESULTS

### Ablation of DJ-1 protects APAP-induced acute liver injury and mortality in mice

**Figure 1:**
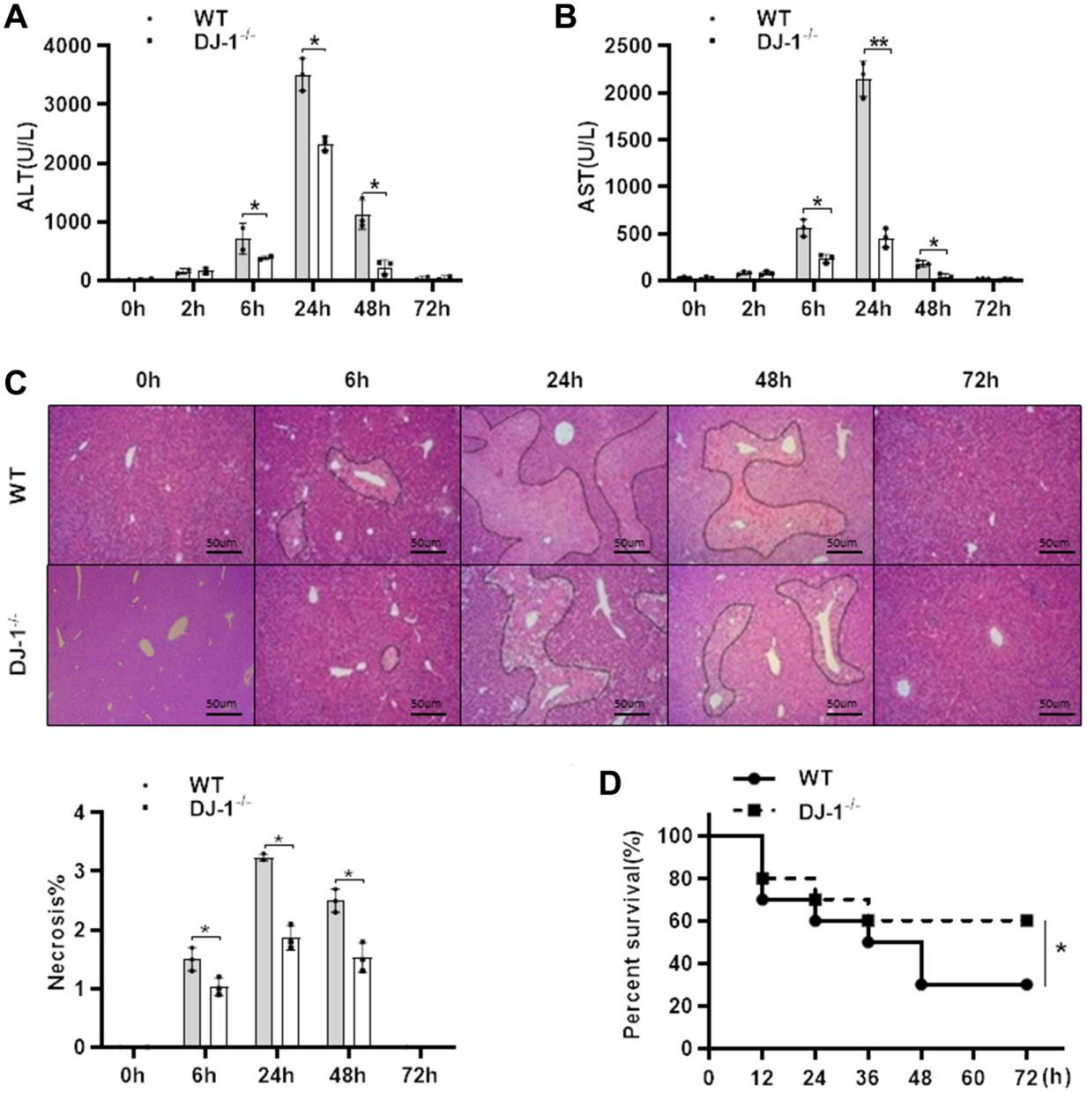
**Ablation of DJ-1 protects APAP-induced acute liver injury and mortality in mice.** Fasted WT and DJ-1^−/−^ mice were intraperitoneal injected with a single dose 300 mg/kg of APAP. Serum levels of ALT (**A**) and AST (**B**) were shown at the indicated time points after APAP injection (*n* = 4–6). (**C**) Representative images of HE staining of liver tissues of WT and DJ^−/−^ mice at the indicated time points after APAP intraperitoneal injection (origin magnification ×100). (**D**) Survival rate of WT and DJ-1^−/−^ mice after APAP (500 mg/kg) administration (*n* = 10 in each group). Data are shown as means ± SD, ^*^P < 0.05.

To investigate the role of DJ-1 in the pathogenesis APAP-induced liver injury, a classic hepatotoxic mouse model was used. Fasted wild type (WT) and DJ-1 deficient (DJ-1^−/−^) mice were intraperitoneal injected with a single dose of 300 mg/kg of APAP. Compared to WT mice, DJ-1^−/−^ mice were more resistant to APAP-induced hepatotoxicity at indicated time points, which exhibited markedly alleviative alanine aminotransferase (ALT) and aspartate aminotransferase (AST) levels (Figure 1A, 1B) and less necrosis area of the liver tissues (Figure 1C) in the first 6 h to 48 h after the intraperitoneal injection of APAP. By intraperitoneal injecting with a single dose of 500 mg/kg of APAP to evaluate the survival rates of both groups of mice, DJ-1^−/−^ mice showed a significantly lower mortality rate relative to WT mice (Figure 1D). These findings suggested that DJ-1 is positively involved in the APAP-induced hepatotoxicity and DJ-1 deficiency could improves APAP-induced liver injury and mortality.

### DJ-1 deficiency alleviates the inflammatory response to APAP-overdose

**Figure 2:**
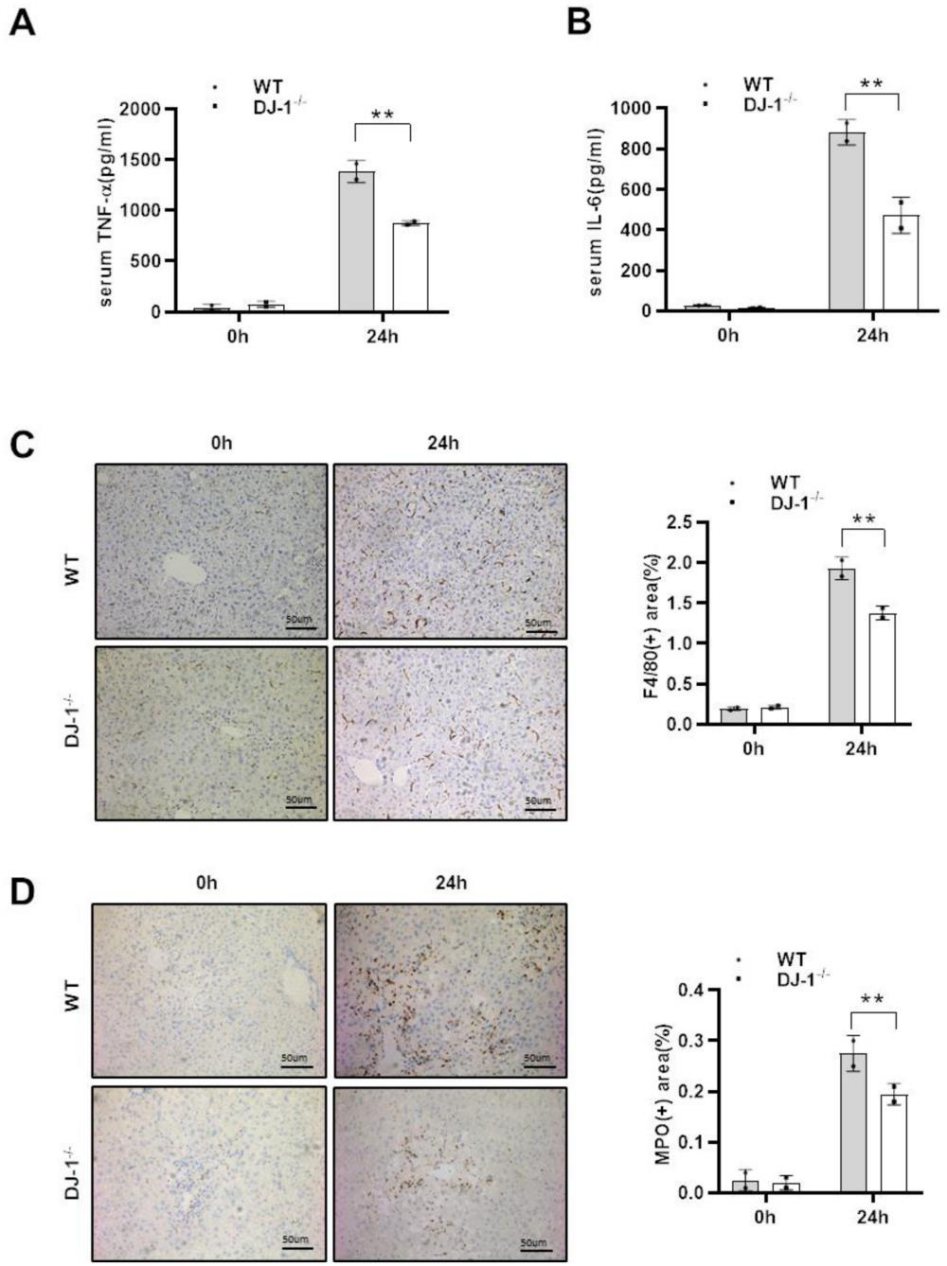
**DJ-1 deficiency attenuates the inflammatory response to APAP overdose.** Fasted WT and DJ-1^−/−^ mice were intraperitoneal injected with a single dose of 300 mg/kg of APAP. Serum TNF-α (**A**) and IL-6 (**B**) levels after APAP injection were measured by ELISA (*n* = 4–6). (**C**) Immunohistochemistry staining of hepatic F4/80 after APAP treatment. Representative images are shown (origin magnification ×100) (*n* = 4–6). (**D**) Immunohistochemistry of hepatic MPO after APAP treatment. Representative images are shown (origin magnification ×100) (*n* = 4–6). Data are shown as means ± SD, ^*^P < 0.05; ^**^P < 0.01.

As shown that there were significant differences in hepatocyte damage between the two mice groups, and given that necrotic hepatocytes can result in inflammatory responses activation to subsequently intensify liver damage after APAP injection, we assessed whether DJ-1 deficiency affects the inflammatory response to APAP-induced liver injury at 24 h (the highest levels of ALT and AST and the largest area of necrosis is detected compared with the other time points). As shown in Figure 2A and 2B, there was significantly less serum levels of tumor necrosis factor-α (TNF-α) and IL-6, which were considered as critical pro-inflammatory cytokines, was observed in DJ-1^−/−^ mice compared to that in WT mice at the indicated time point after APAP treatment. Consistently, IHC staining of F4/80 and MPO in the liver also showed less macrophages and neutrophils infiltration in the liver of DJ-1^−/−^ mice at 24 h after APAP treatment (Figure 2C, 2D). These results suggested that ablation of DJ-1 alleviates APAP induced liver injury and inflammatory responses. However it is still not clear that the protective effects of ablation of DJ-1 is through modulating hepatocytes or inflammatory cells response.

### DJ-1 deficiency does not affect APAP metabolism and mitochondrial dysfunction both *in vivo* and *in vitro*

**Figure 3:**
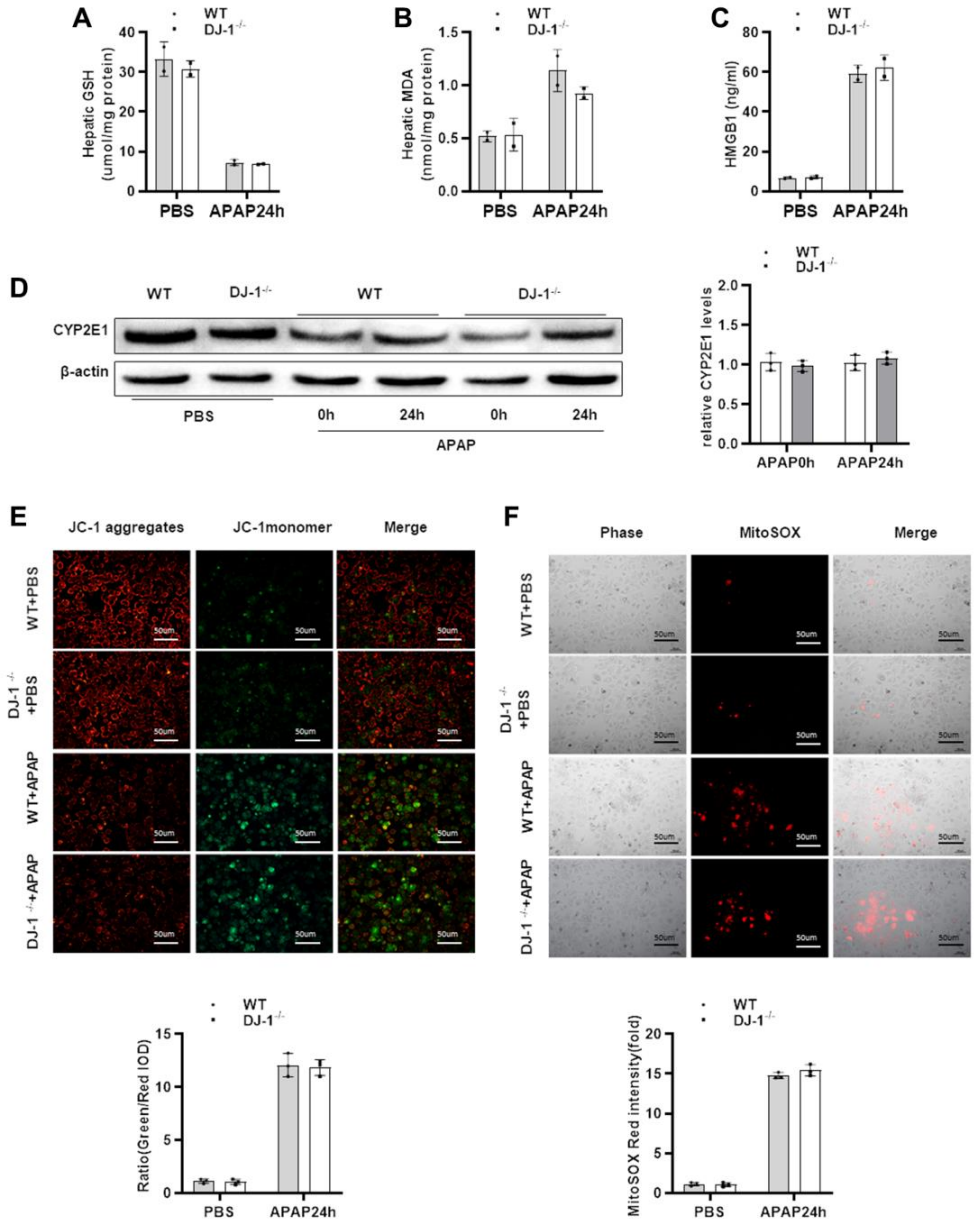
**DJ-1 deficiency does not affect APAP metabolism and mitochondrial dysfunction both *in vivo* and *in vitro*.** Fasted WT and DJ-1^−/−^ mice were intraperitoneal injected with a single dose of 300 mg/kg of APAP. (**A**) GSH levels after APAP treatment (*n* = 3–5). (**B**) MDA levels after APAP treatment (*n* = 3–5). (**C**) HMGB1 levels after APAP treatment (*n* = 3–5). (**D**) Western blot analysis of CYP2E1 in liver tissues after APAP treatment. B-actin was used as control. (**E**) Isolated primary WT and DJ-1^−/−^ hepatocytes were starved and treated with APAP. Representative images of mitochondrial membrane potentials in primary hepatocytes 6h after APAP treatment evaluated using JC-1 (origin magnification ×100) (*n* = 3). (**F**) Representative images of mitochondrial ROS in primary hepatocytes 6h after APAP treatment evaluated with the MitoSOX Red probe (origin magnification ×100) (*n* = 3). Data are shown as means ± SD.

In order to investigate whether DJ-1 modulate hepatocytes function during APAP treatment, we checked whether hepatic DJ-1 deficiency could affect APAP metabolism. We first measured hepatic GSH, MDA, HMGB1 levels at 24 h after APAP treatment which are closely related with APAP caused direct hepatocytes injury. As shown in Figure 3A–3C, there were no significant differences between DJ-1^−/−^ and WT mice. Cytochrome P450 2E1 (CYP2E1) plays a central role in APAP metabolism, and subsequently generates metabolic products, such as mitochondrial reactive oxygen species (ROS), caused mitochondrial dysfunction in the early stage of the pathogenesis of APAP-induced hepatotoxicity [4, 9]. We also measured CYP2E1 expression level in the liver. There was no significant difference between DJ-1^−/−^ and WT mice (Figure 3D). These findings suggest that DJ-1 may not affect APAP induced hepatocytes injury. To further confirm this, APAP-induced mitochondrial dysfunction of the primary hepatocytes which were isolated from DJ-1^−/−^ mice and WT mice were investigated. Primary hepatocytes were starved and treated with 5 mM APAP *in vitro* (treated with PBS as control), and mitochondrial dysfunction of both groups was evaluated by the measurement of mitochondrial membrane potential ΔΨm (JC-1 fluorescent dye) and mitochondrial ROS (MitoSOX Red dye) 24 h after APAP treatment, respectively. As demonstrated by JC-1 and MitoSOX Red signals after APAP treatment, hepatocytes from DJ-1^−/−^ mice showed no different mitochondrial dysfunction compared to that in WT mice (Figure 3E, 3F).

### Hepatic DJ-1 deficiency has no protective effect on APAP-induced liver injury

**Figure 4:**
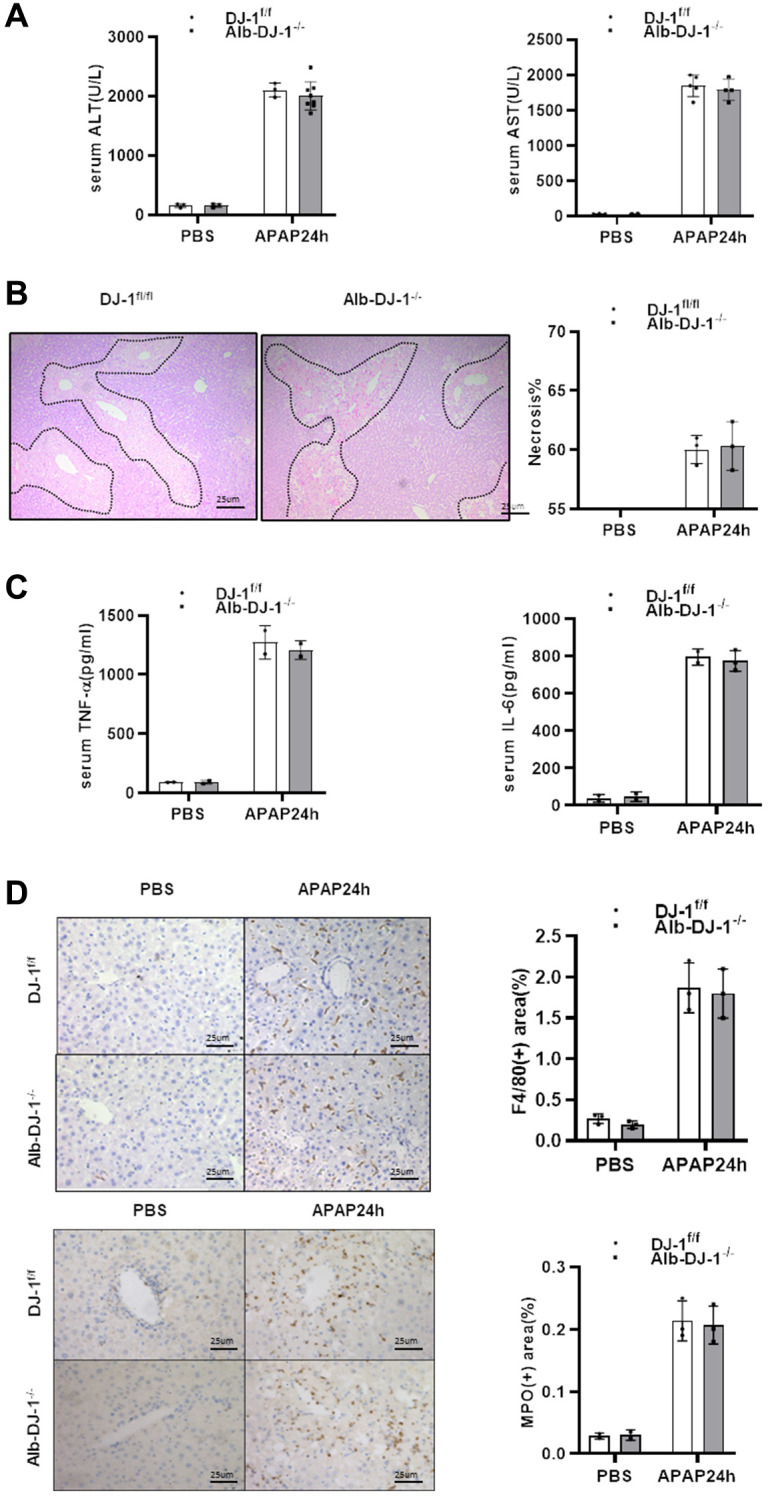
**Hepatic DJ-1 deficiency has no protective effect on APAP-induced liver injury.** Fasted DJ-1^fl/fl^ and Alb-DJ-1^−/−^ mice were intraperitoneal injected with a single dose of 300mg/kg of APAP. (**A**) Serum levels of ALT and AST 24h after APAP treatment (*n* = 4–6). (**B**) Representative images of hepatic HE staining of DJ-1^fl/fl^ and Alb-DJ-1^−/−^ mice 24 h after APAP challenge (origin magnification ×200). (**C**) Serum levels of TNF-α and IL-6 24 h after APAP treatment (*n* = 4–6). (**D**) Immunohistochemistry of hepatic F4/80 and MPO with APAP treatment. Representative images are shown (origin magnification ×200) (*n* = 4–6). Data are shown as means ± SD.

Given that DJ-1 could not directly modulate APAP induced hepatocytes mitochondrial dysfunction and injury, we further generated hepatocyte-specific DJ-1-knockout (Alb-DJ-1^−/−^) mice by crossing DJ-1fl/fl mice with AlbCre+ mice (Supplementary Figure 1). We investigated whether hepatic DJ-1 deficiency could affect APAP-induced liver injury. As evidenced by the serum levels of ALT and AST, Alb-DJ-1^−/−^ mice showed equivalent liver injury in response to APAP-induced hepatotoxicity compared to DJ-1^fl/fl^ control mice (Figure 4A). Consistently, there were no statistical differences in hepatic necrosis areas between DJ-1^fl/fl^ control mice and Alb-DJ-1^−/−^ mice (Figure 4B). In addition, there were analogous serum levels of proinflammatory cytokines, such as IL-6 and TNF-α, between DJ-1^fl/fl^ and Alb-DJ-1^−/−^ mice (Figure 4C). Furthermore, the results of immunohistochemical staining showed that there were no significant differences in hepatic macrophages and neutrophils infiltration in both genotype mice groups (Figure 4D). All these findings clearly indicated that DJ-1 deficiency in hepatocytes does not affect APAP-induced liver injury.

### Myeloid DJ-1 deficiency protects the liver from APAP induced liver injury

**Figure 5:**
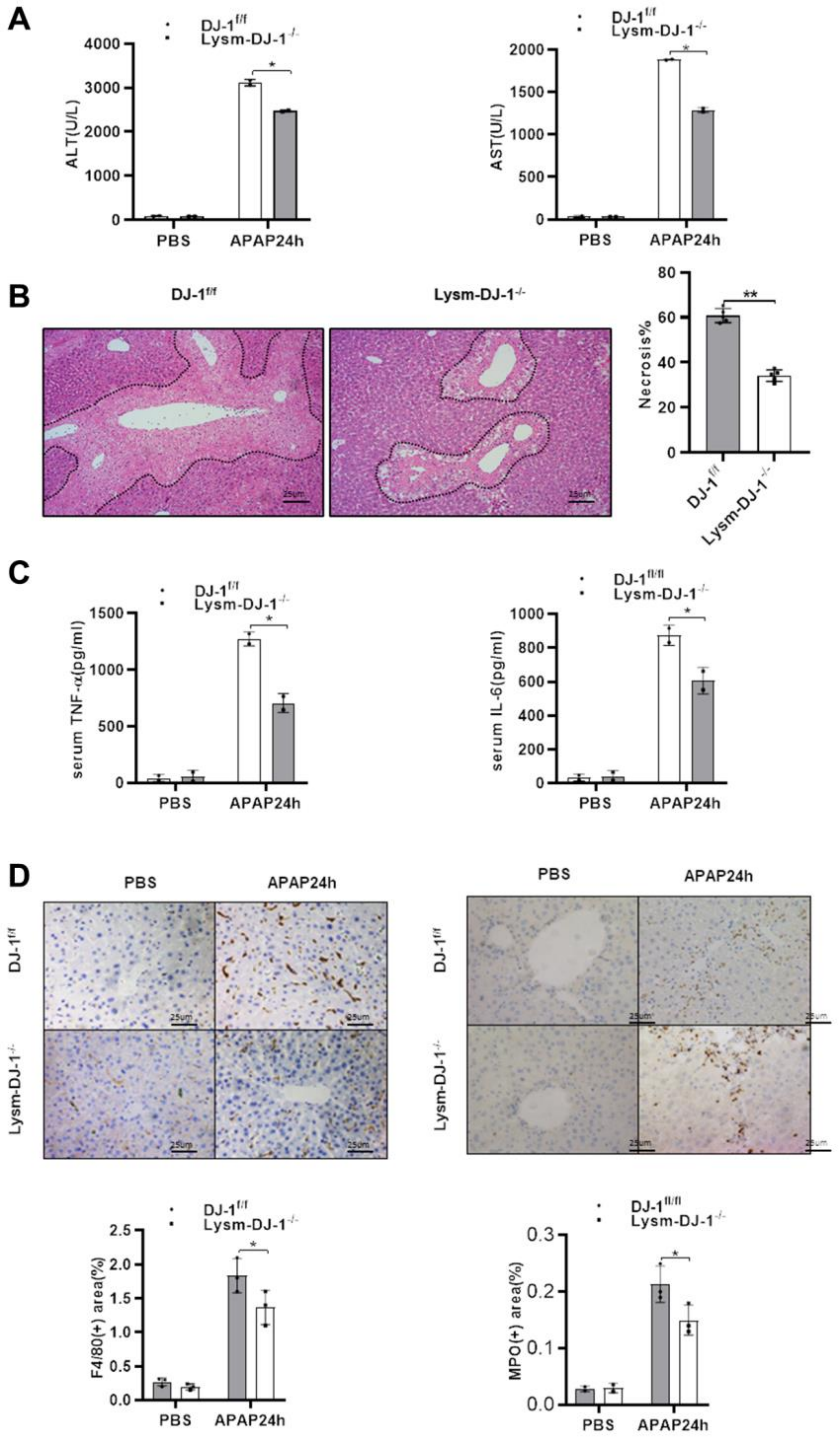
**Myeloid DJ-1 deficiency protects the liver from APAP induced liver injury.** Fasted DJ-1^fl/fl^ and Lysm-DJ-1^−/−^ mice were intraperitoneal injected with a single dose of 300mg/kg of APAP. (**A**) Serum levels of ALT and AST 24h after APAP treatment (*n* = 4–6). (**B**) Representative images of hepatic HE staining of DJ-1^fl/fl^ and Lysm-DJ-1^−/−^ mice 24 h after APAP challenge (origin magnification ×200). (**C**) Serum levels of TNF-α and IL-6 24 h after APAP treatment (*n* = 4–6). (**D**) Immunohistochemistry of hepatic F4/80 and MPO with APAP treatment. Representative images are shown (origin magnification ×200) (*n* = 4–6). Data are shown as means ± SD, ^*^P < 0.05; ^**^P < 0.01.

Having demonstrated that hepatic DJ-1 depletion does not protect against APAP-induced hepatotoxicity, we investigated whether DJ-1 deficiency in innate immune cells could prevent from the liver injury. By crossing DJ-1^fl/fl^ mice with LysmCre+ mice, specific myeloid DJ-1 knock-out mice were generated (Supplementary Figure 1). Compared to DJ-1^fl/fl^ control mice, serum levels of ALT and AST, which represented the severity of the hepatic injury, were significantly decreased in Lysm-DJ-1^−/−^ mice (Figure 5A). Liver necrosis areas were also remarkably decreased in Lysm-DJ-1^−/−^ mice (Figure 5B). In addition, the serum levels of proinflammatory cytokines (TNF-α and IL-6), were significantly lower in Lysm-DJ-1^−/−^ mice compared to control mice (Figure 5C). Correspondingly, the results of immunohistochemical staining showed that there were no significant differences in macrophages and neutrophils infiltration in the liver in both genotype mice groups (Figure 5D). These findings indicated that DJ-1 deficiency in innate immune cells plays a protective role in APAP-induced hepatotoxicity.

### Myeloid DJ-1 deficiency attenuates APAP induced hepatic inflammatory reaction via less ROS production and chemokines induction

**Figure 6:**
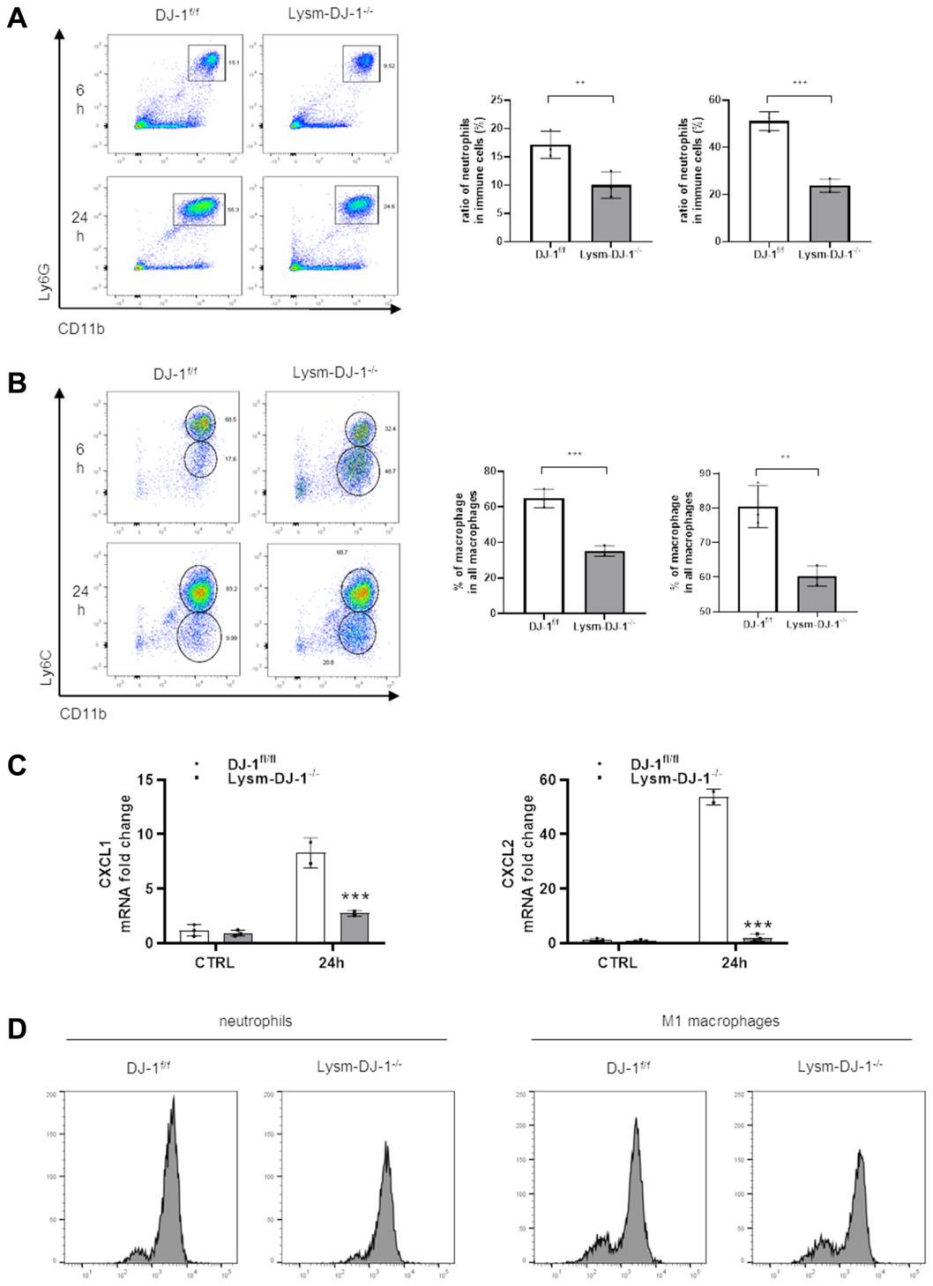
**Myeloid DJ-1 deficiency attenuates APAP induced hepatic inflammatory reaction via less ROS production and chemokines induction.** Fasted DJ-1^fl/fl^ and Lysm-DJ-1^−/−^ mice were intraperitoneal injected with a single dose of 300 mg/kg of APAP. Quantification of Ly6G^+^CD11b^+^ neutrophils (**A**) and Ly6C^+^CD11b^+^ macrophages (**B**) by flow cytometry (*n* = 4). (**C**) Quantification of CXCL1 and CXCL2 expression in liver tissue 24 h after APAP challenge (*n* = 5–8). (**D**) Quantification of cellular ROS levels by oxidized DCFDA and flow cytometry in neutrophils and macrophages isolated from DJ-1^fl/fl^ and Lysm-DJ-1^−/−^ mice (*n* = 4). Data are shown as the means ± SD, ^*^P < 0.05; ^**^P < 0.01; ^***^P < 0.001; ^****^P < 0.0001.

Given that DJ-1 is essential for ROS production in proinflammatory cells [24]. In our study there were no protective effects in hepatocyte-specific DJ-1-knockout (Alb-DJ-1^−/−^) mice, whereas there were significant protective effects in myeloid DJ-1 knock-out mice, which clearly demonstrated that the protective function of DJ-1 deficiency was through modulating innate immune cells responses during APAP induced liver injury. To further test the inflammatory responses, we isolated intrahepatic immune cells from DJ-1^fl/fl^ and Lysm-DJ-1^−/−^ mice at 6h or 24 h after APAP treatment. By measuring these isolated intrahepatic immune cells, it was shown that there was a decreased population of Ly6G^+^CD11b^+^ cells (neutrophils) and Ly6C^high^CD11b^+^ cells (M1 macrophages) in Lysm-DJ-1^−/−^ mice compared to those in DJ-1^fl/fl^ control mice (Figure 6A, 6B and Supplementary Figure 2). Correspondingly, gene expression of the chemokines (CXCL1 and CXCL2) was significantly lower in Lysm-DJ-1^−/−^ mice compared to control mice (Figure 6C). As one of the main activation markers of immune cells, the measurement of the isolated intrahepatic immune cells showed a decreased ROS production in neutrophils and M1 macrophages of Lysm-DJ-1^−/−^ mice, respectively (Figure 6D). These results clearly demonstrated that DJ-1 deficiency caused lower inflammatory response via less ROS production and infiltration of proinflammatory cells, thus alleviate the sterile inflammation in hepatic necrotic areas, which may result in the improved liver injury in Lysm-DJ-1^−/−^ and DJ-1^−/−^ mice induced by APAP overdose.

## DISCUSSION

In this present study, by adopting the classic mouse model of APAP-induced hepatotoxicity, we demonstrated that DJ-1 deficiency protected against APAP-induced acute liver injury and alleviative proinflammatory cells infiltration and proinflammatory cytokines induction. Mechanistically, we found that DJ-1 deficiency does not affect CYP2E1 expression and mitochondrial dysfunction, therefore there were no protective effects in hepatic DJ-1 deficiency mice in APAP-induced hepatotoxicity model. Additionally, myeloid DJ-1 deficiency improved liver injury by impairing hepatic proinflammatory response. Consistently, there were less pro-inflammatory immune cells and less ROS production and chemokines induction in Lysm-DJ-1^−/−^ mice, which indicated that DJ-1 deficiency through decreasing inflammatory response and could be a potential therapeutic and/or prophylactic target for APAP-induced acute liver injury.

DJ-1 is a multi-functional protein involved in the progression of various diseases, and a previous study of quantitative proteome profiling in acetaminophen-treated three-dimensional liver microtissues has shown that DJ-1 was changes during APAP treatment [28]. To verify the relationship between DJ-1 and APAP-induced hepatotoxicity in mice, mice were intraperitoneal injected with a dose of APAP at different indicated time points and DJ-1 deficiency alleviated the severity of the liver injury and the mortality rate of the mice was confirmed. To further investigate the effects of DJ-1 in APAP induced hepatotoxicity, we also evaluated the inflammatory response. In previous studies, neutrophils and macrophages infiltration into the liver has been considered as an important character in APAP-induced acute liver injury/failure, because of the excessive ROS and proinflammatory cytokines production of neutrophils to induce secondary liver injury [29, 30]. In the present study, there was significantly less induction of tumor necrosis factor-α (TNF-α) and IL-6 in DJ-1^−/−^ mice, and there was less hepatic infiltration of macrophages and neutrophils in DJ-1^−/−^ mice. These results were consistent with a recent study which showed that recombinant DJ-1 protein induces the production of various inflammatory cytokines in macrophages [31]. In conclusion, we confirmed that DJ-1 deficiency protected APAP-induced hepatotoxicity and also proinflammatory cell infiltration and proinflammatory cytokine induction.

Given that DJ-1 has been demonstrated to be expressed in hepatocytes as well as in immune cells previously [23, 32], and since hepatocytes and immune cells are two main cell sources involved in APAP-induced hepatotoxicity, we wondering that hepatic and/or myeloid-derived DJ-1 were responsible for APAP-induced hepatotoxicity. In our current study, we found that hepatic DJ-1 deficiency does not affect APAP metabolism and mitochondrial dysfunction in hepatocytes, therefore there were also no beneficial effects on APAP-induced hepatotoxicity. In accordance with these findings, our *in vivo* studies confirmed that hepatic DJ-1 deficiency could not alleviate the severity of the liver injury and inflammatory response. Furthermore, our data clearly defined that myeloid-derived DJ-1 deficiency could protect against APAP-induced hepatotoxicity, suggesting that the modulation of DJ-1 in immune cells may be responsible for the aggravation of APAP-induced liver injury.

Our group have been investigated a series of functions of DJ-1 in modulating inflammatory responses in both liver diseases and systemic inflammatory models [24, 32, 33], especially in myeloid cells we demonstrated that DJ-1 is essential for the activation of innate immune cells through modulating ROS production. In the present study, we demonstrated that DJ-1 deficiency in myeloid cells showing less proinflammatory cells infiltration and activation. There were less ROS levels and cytokines production further confirmed the lower inflammatory response in DJ-1 deficiency inflammatory cells. Thus, DJ-1 plays a role in decreasing APAP-induced aseptic inflammation and protecting against liver injury.

In conclusion, our study clearly demonstrated that DJ-1 deficiency in innate immune cells plays a critical role in the pathogenesis of APAP-induced acute liver injury/failure via decreasing proinflammatory responses. These findings could open up a new insight into the treatment and/or prevention of APAP-induced acute liver failure.

## MATERIALS AND METHODS

### Animals and treatment

All animal-involved experiments were reviewed and approved by the Institutional Animal Care and Use Committees (IACUC) of Shuguang Hospital affiliated to Shanghai University of Chinese Traditional Medicine. DJ-1 knockout (DJ-1^−/−^) mice (B6.Cg-Spp1tm1Blh/J) were purchased from the Jackson Laboratory (Bar Harbor, ME, USA). DJ-1^fl/fl^ mice were generated and AlbCre^+^, LysmCre^+^ mice were purchased from Shanghai Biomodel Organism Science and Technology Development Co. Ltd (Shanghai, China). Male mice aged 6–8 weeks were used for study and all mice were fed under SPF conditions with a 12-hour diurnal cycle. Mice were firstly fasted for 15–17 hours, and then intraperitoneal injected with 300 mg/kg of APAP (#908312, Sigma-Aldrich, St. Louis, MO, USA) for functional experiment and 500 mg/kg for mortality rate experiment.

### Biochemical measurement

Mice were sacrificed, sterilized with alcohol, and blood was collected through direct enucleation and centrifuged at 12000 rpm for 5 min. Microplate test kits (C009-2-1 and C010-2-1, Nanjing Jiancheng Bioengineering Institute, Nanjing, China) were used for the measurement of serum ALT and AST levels according to the manufacturer’s protocols.

### Enzyme-linked immunosorbent assay

Serum levels of IL-6, TNF-α (EMC004.96 and EMC102a.96, Neo Bioscience Technology, Shenzhen, China) were measured by Mouse ELISA Kits according to the manufacturer’s protocols. Reagents, samples, and standards were prepared as instructed. After adding 50 μl standards and serum samples per well, the 96-well plate was incubated for 2 hours at 37°C. Then the liquid was removed and washed. And then 100 μl of Biotin antibody was added to each well before incubating for 1 h at 37°C, and followed by washing the plate 5 times with a washing buffer. 100 μl of HRP-avidin was added to each well, incubated for 1 hour at 37°C, and washed 5 times. After adding 90 μl TMB substrate to each well, the plate was protected from light and incubated for 20 minutes at 37°C. Color formation was stopped by 50 μl stop solution, and the optical density (OD) value was read at wavelength of 450 nm on a plate microplate reader within 5 minutes. Corresponding concentrations were converted from OD values according to the standard curve. Each serum sample was tested twice and the average value was taken for analysis.

### Histological analysis and immunohistochemistry

Mice were sacrificed, alcohol sterilized, and liver tissues were harvested and fixed for at least 24 hours in 4% paraformaldehyde. The tissues were then embedded in paraffin, sliced into 5 μm-thick sections and placed on the glass slides. The sections were subsequently stained with hematoxylin and eosin following classic protocols. For immunohistochemistry (IHC), after the tissues were dewaxed and dehydrated with xylene and ethanol, washed with water, the tissues repaired with sodium citrate, and incubated with 3% hydrogen peroxide to inhibit the activity of endogenous peroxidase. Then, the tissues were blocked with 5% BSA at 37°C for 1 h. Then 100 μl primary antibodies against mouse MPO (1: 100, BioLegend, San Diego, CA, USA) or mouse F4/80 (1: 100, Abcam, Cambridge, UK) were added, and the tissues were placed in a 4°C wet box overnight. The next day, the corresponding horseradish peroxidase conjugated secondary antibody was added to the tissues for incubation at room temperature for 2 h. Signals were developed by using diaminobenzidine as a substrate for 2 min. Images were then acquired under an optical microscope (×100/×200).

### Hepatocyte isolation

As previously mentioned, primary hepatocyte isolation was conducted [33]. The isolated hepatocytes were counted and 2 × 10^6^ cells were cultured with 5ml medium (DMEM supplemented with 10% fetus bovine serum and 1% penicillin-streptomycin (Gibco, Grand Island, NY, USA)) in 6 cm dishes, 5 × 10^5^ cells/well with 3 ml medium in 6-well plates, or 5 × 10^4^ cells/well with 1 ml medium in 24-well plates.

### Flow cytometry analysis

Hepatic immune cells were isolated as we described previously [33]. Collected immune cells were stained with APC-CY7-conjugated anti-CD45 (#A15395, eBioscience, San Diego, CA, USA), PerCP-CY5.5-conjugated anti-CD11b (#45-0112-80, eBioscience, San Diego, CA, USA), PE-conjugated anti-F4/80(#MF48004-3, eBioscience, San Diego, CA, USA), BV421-conjugated anti-Ly-6G (#9668-82, eBioscience, San Diego, CA, USA) and APC-conjugated anti-Ly-6C (#17-5932-82, eBioscience, San Diego, CA, USA). The purity of these isolated immune cells was over 90%.

### Cellular ROS quantification by flow cytometry

The intracellular ROS levels were measured by dichlorodihydrofluorescein diacetate (DCFDA). In short, the cells were incubated in 5 mM DCFDA (#D399, Thermo Fisher Scientific, Waltham, MA, USA) for 1 h at 37°C. The fluorescence intensity indicating the ROS activity was detected with flow cytometry.

### Western blot

Liver tissues or cell samples were homogenized with RIPA buffer (#89901, Thermo Scientific, Rockford, IL) containing a protease inhibitor cocktail (#87786, Caliche, Raleigh, NC, USA) and phosphatase inhibitor cocktail (HY-K0021; Med Chen Express). The Pierce BCA Protein Assay Kit (#23227, Thermo Fisher Scientific) was used to measure total protein concentrations. Protein samples (40 μg) were separated on a 10% SDS–PAGE gel. The gel was transferred to a nitrocellulose membrane. The NC membrane was blocked with 5% non-fat milk for one hour at room temperature, and then incubated with primary antibodies against indicated antigens at 4°C overnight under shaking conditions. The membrane incubation with the corresponding secondary antibodies coupled with horseradish peroxidase for one hour at room temperature then. The Cheviot XRS+ System with Image Lath Software (Bio-Rad) was used for blotting signal detection and quantification.

### Statistical analysis

The data is represented in the form of means ± SD. Statistical significance was estimated by using two-tailed, unpaired or paired Student’s *t* test. By using the log-rank (Mantel-Cox) test, survival curves were contrasted. In all statistical analysis, *P*-value < 0.05 was considered to indicate a statistically significant difference.

### Ethics approval and consent to participate

All animal-involved experiments were reviewed and approved by the Institutional Animal Care and Use Committees (IACUC) of Shuguang Hospital affiliated to Shanghai University of Chinese Traditional Medicine.

## Supplementary Materials

Supplementary Figures
