# Supplementary material for: Myeloid DJ-1 deficiency protects acetaminophen-induced acute liver injury through decreasing inflammatory response
Source: Aging (Albany NY). 2021 Jul 21;13(14):18879–93. doi: 10.18632/aging.203340 (PMC8351717; doi:10.18632/aging.203340)
Supplement: Supplementary Figures [file aging-13-203340-s001.pdf]

SUPPLEMENTARY FIGURES

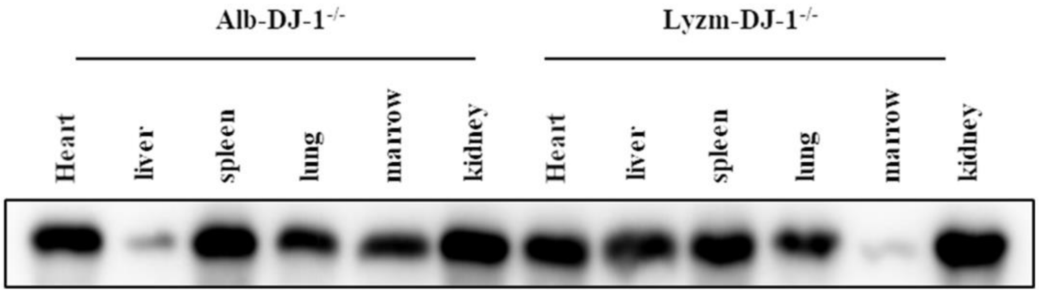

Supplementary Figure 1. Protein expression of various tissues in Alb-DJ-1$^{-/-}$ and Lyzm-DJ-1$^{-/-}$ mice.

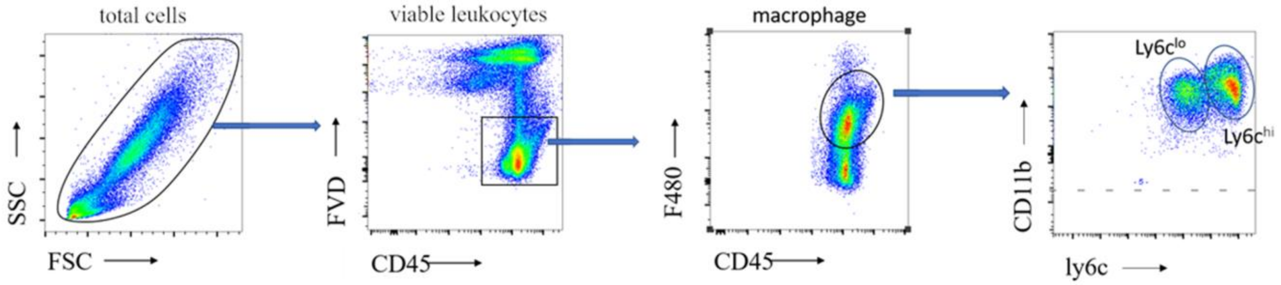

Supplementary Figure 2. Gate selection of two subtypes of macrophages by flow cytometry.
